# Familiarity with Own Population’s Appearance Influences Facial Preferences

**DOI:** 10.1007/s12110-017-9289-8

**Published:** 2017-05-18

**Authors:** Carlota Batres, Mallini Kannan, David I Perrett

**Affiliations:** School of Psychology and Neuroscience, University of St Andrews, Fife, KY16 9JP UK

**Keywords:** Preferences, Faces, Adiposity, Familiarity, Cross-cultural analyses

## Abstract

Previous studies have found that individuals from rural areas in Malaysia and in El Salvador prefer heavier women than individuals from urban areas. Several explanations have been proposed to explain these differences in weight preferences but no study has explored familiarity as a possible explanation. We therefore sought to investigate participants’ face preferences while also examining the facial characteristics of the actual participants. Our results showed that participants from rural areas preferred heavier-looking female faces than participants from urban areas. We also found that the female faces from the rural areas were rated as looking heavier than the female faces from the urban areas. Our findings are consistent with the hypothesis that familiarity may be contributing to the differences found in face preferences between rural and urban areas given that people from rural and urban areas are exposed to different faces.

Previous studies have found that individuals from rural areas prefer heavier women than individuals from urban areas (Batres and Perrett 2014; Swami and Tovée 2005). For example, Swami and Tovée (2005) found that male and female participants from a rural area in Malaysia preferred female bodies with higher body mass indices (BMIs) than participants from urban areas did. Similarly, in El Salvador, Batres and Perrett (2014) found that male and female participants from a rural area found faces of heavier women more attractive than participants from an urban area.

Several explanations have been proposed to explain these differences in weight preferences observed between people from rural and urban areas. One explanation is that there are differing optimal weights for different environments given that BMI is closely related to health (Lake et al. 1997) and fertility (Frisch 1988). In rural environments with less certain food availability, women with higher BMIs may be better equipped to survive and reproduce (Brown and Konner 1987) and therefore preferences for such women could be adaptive.

A second explanation for the differences in weight preferences between people from rural and urban areas is that of urbanization. For instance, Scott et al. (2014) found that urbanization is the best predictor of facial stereotyping out of a range of other potentially relevant predictors. They proposed that exposure to a large number of unfamiliar faces in urban areas may be relevant to the development of WEIRD (Western, Educated, Industrialized, Rich, and Democratic; Henrich et al. 2010) preferences and perceptions. Other research has indeed found that urbanization predicts changes in basic perceptual processes (Caparos et al. 2012; Linnell et al. 2013). In addition, people in urban areas have higher levels of media exposure (Chan and McNeal 2006) and research has shown that the media promotes beauty ideals of low body weight in women (Katzmarzyk and Davis 2001; Voracek and Fisher 2006). For example, Voracek and Fisher (2006) found that starring roles in movies produced by a leading European adult media company were more likely to be played by actresses with low BMIs. Given that exposure to media is often greater in urban areas than rural areas (Chan and McNeal 2006), higher weight preferences among rural participants may be due to their lower levels of exposure to such beauty ideals.

In this study, we investigate the possibility of a third explanation: people from rural and urban areas may have a different visual diet of faces and, if so, familiarity could be contributing to their weight preferences. Indeed, research has found that exposure to a certain population of faces increases the attractiveness of similar faces (Cooper and Maurer 2008; Saxton et al. 2009). For instance, girls who attend single-sex schools prefer more feminized male and female faces than girls who attend mixed-sex schools (Saxton et al. 2009). Familiarity can also be manipulated experimentally, with exposure to certain facial features leading to a preference for faces with similar facial features later on (Cooper and Maurer 2008). The after-effects of such exposure can last for minutes (Cooper and Maurer 2008), days (Carbon et al. 2007), or weeks (Carbon and Ditye 2011).

We thus examine whether familiarity could be responsible for the findings that individuals from rural areas prefer heavier women than individuals from urban areas in Malaysia (Swami and Tovée 2005) and El Salvador (Batres and Perrett 2014). We tested participants from rural and urban areas with regard to their preferences for facial adiposity (i.e., the perception of weight in faces; Coetzee et al. 2009). We then took facial images of these participants and had another set of participants rate their faces on how underweight/overweight they appeared. Based on previous research (Swami and Tovée 2005; Batres and Perrett 2014), we predicted that the rural participants would find female faces with higher levels of adiposity more attractive than the urban participants in both Malaysia and El Salvador. We also predicted that the faces of the women from the rural areas would be rated as heavier than the faces of the women from the urban areas since adverse environments with attendant stresses can predispose fat accumulation (Chrousos 2009). Previous research has not consistently found a preference for male faces with higher levels of adiposity in rural areas (Batres and Perrett 2014) and therefore, we predicted that there would be no difference between rural and urban participants in adiposity preferences for male faces. Lastly, we also predicted that there would be no difference in weight ratings between the faces of the men from the rural areas and the faces of the men from the urban areas in both Malaysia and El Salvador.

## Methods: Study 1

### Materials

Facial images of Caucasian men and women photographed facing forward, under constant camera and lighting conditions, with neutral expressions, no adornments, and closed mouths were selected from an online database (3D.SK 2012). These images were delineated with 189 points using Psychomorph, a custom software (Tiddeman et al. 2001), and aligned to a standard inter-pupillary distance (Rowland and Perrett 1995). Ten composite images (5 male and 5 female) were created (each averaging 3 original faces merged together) and masked with a black oval around the head to occlude clothes.

**Fig. 1:**
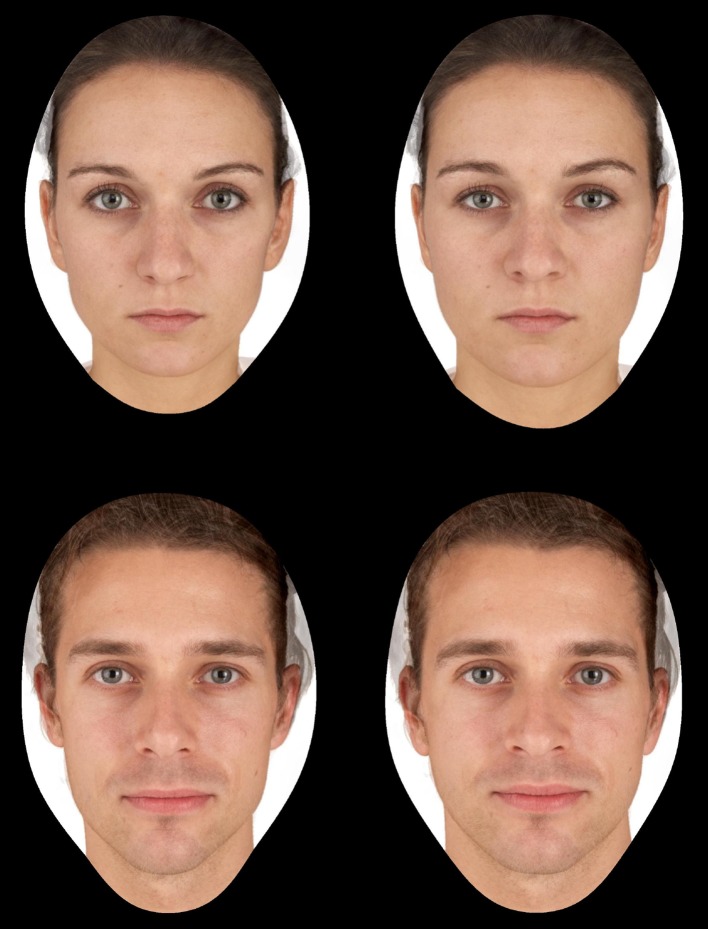
Example of facial stimuli. One of the female adiposity pairs (*top*) and one of the male adiposity pairs (*bottom*). The faces on the left correspond to a low-BMI face shape and the faces on the right correspond to a high-BMI face shape

Face prototypes were then created to use for transforming the composites in adiposity. The male adiposity prototypes were generated by separately averaging male faces with a low BMI (*M* = 22.19 kg/m^2^, SD = 2.52; *M*_age_ = 25.10 years, SD = 3.96) and male faces with a high BMI (*M* = 26.47 kg/m^2^, SD = 3.27; *M*_age_ = 24.80 years, SD = 3.77). The female adiposity prototypes were generated by separately averaging female faces with a low BMI (*M* = 17.85 kg/m^2^, SD = 0.80; *M*_age_ = 22.70 years, SD = 3.56) and females faces with a high BMI (*M* = 24.06 kg/m^2^, SD = 6.34; *M*_age_ = 23.40 years, SD = 4.50) (for details see Batres et al. 2015). The prototypes were then used to create transforms of the 10 composite faces with ±50% of the shape difference while holding texture and color constant. This resulted in a total of 10 pairs of faces, where 5 pairs were of women made up of a low-BMI and a high-BMI face shape and 5 pairs were of men made up of a low-BMI and a high-BMI face shape (Fig. 1).

### Procedures and Participants

Ethical approval was received from the University of St Andrews Ethics Board. Participants were recruited through word-of-mouth and they provided written consent after being read the consent information. They were first given a stack of laminated sheets that consisted of 5 pairs of male faces and 5 pairs of female faces. Each laminated sheet consisted of a pair of faces; the faces that appeared on the left/right were randomized. Participants pointed at the face from each pair they considered to be the most attractive. There was no time limit. The participants then completed a short questionnaire that requested demographic information, such as their sex, age, and where they were living. Lastly, their weight (using a digital scale) and height were measured and a standardized photograph of their face (facing forward, with hair pulled back, under constant camera and lighting conditions, with neutral expressions, no adornments, no cosmetics, and closed mouths) was taken. Each participant was paid in local currency (15 ringgit in Malaysia and US $5 in El Salvador) upon completion of the experiment. Forty-three men (*M*_age_ = 20.44 years, SD = 1.75) and 65 women (*M*_age_ = 19.95 years, SD = 1.59) aged 18–24 from Malaysia and 69 men (*M*_age_ = 20.71 years, SD = 1.90) and 83 women (*M*_age_ = 20.46 years, SD = 2.09) aged 18–25 from El Salvador completed the study (see Table 1 for participant information).

**Table 1:** Participant information

	Rural			Urban		
	*N*	*M*_age_ (years)	SD	*N*	*M*_age_ (years)	SD
Malaysia	18 men	20.83	1.79	25 men	20.16	1.70
	24 women	20.13	1.92	41 women	19.85	1.37
El Salvador	38 men	20.66	1.76	31 men	20.77	2.08
	43 women	20.53	2.31	40 women	20.38	1.84

### Country Information

El Salvador was chosen as a test site because one of the researchers is Salvadoran, which reduced the safety risk involved in field testing. A Malaysian researcher was also recruited. Having researchers from the relevant countries allowed the study to be conducted in the native languages of each test site (Malay in Malaysia and Spanish in El Salvador).

Malaysia covers 329,847 km^2^ (Central Intelligence Agency [CIA] 2016). It has a population of 29.9 million, among whom 0.6% fall below the national poverty line (each country determines its own poverty line; World Bank 2014). It has a GDP of $338.1 billion (World Bank 2014), which is made up of 8.9% agriculture, 35.0% industry, and 56.1% services (CIA 2016). The sample areas in Malaysia were Pahang (rural) and Selangor (urban).

El Salvador covers 21,041 km^2^ (CIA 2016). It has a population of 6.1 million, among whom 31.8% fall below the national poverty line (World Bank 2014). It has a GDP of $25.2 billion (World Bank 2014), which is made up of 10.7% agriculture, 25.5% industry, and 63.8% services (CIA 2016). The sample areas in El Salvador were Ahuachapán (rural) and San Salvador (urban).

## Methods: Study 2

### Procedures and Participants

Ethical approval was received from the University of St Andrews Ethics Board. Participants were recruited through MTurk, and they provided consent online after being presented with the consent information. Participants rated the face images collected in Study 1, individually and in random order. Participants rated either the face images from Malaysia or the face images from El Salvador. The faces from the rural and urban populations were intermixed and each face was masked with a black oval around the head to occlude clothes. The images were presented one sex at a time, and participants were instructed to rate how heavy they thought each man/woman was on a 10-point Likert scale (1 = very underweight, 10 = very overweight). The participants then completed a short questionnaire that requested demographic information, such as their sex and age. Each participant was paid $2 dollars through MTurk upon completion of the experiment. Twenty men (*M*_age_ = 29.80 years, SD = 5.36) and 20 women living in the United States (*M*_age_ = 32.90 years, SD = 9.70) successfully rated (i.e., responded “yes” to the question “Were you able to see and rate all images successfully?”) the faces from Malaysia on weight. Twenty men (*M*_age_ = 36.30 years, SD = 10.10) and 19 women (*M*_age_ = 33.58 years, SD = 9.61) living in the United States successfully rated the faces from El Salvador on weight.

## Results: Study 1

### Malaysia

Adiposity preferences were calculated by taking the percentage of faces high on the selected trait across the 5 pairs of male faces and the 5 pairs of female faces. Independent samples *t*-tests revealed no significant effect of sex of participant on preferences (*p* > 0.882 for all analyses). Therefore, for all subsequent analyses, data from male and female participants were aggregated. One sample *t*-tests revealed that the selected faces were significantly different from chance in the urban sample (*p* < 0.022 for all analyses) but not in the rural sample (*p* > 0.379 for all analyses). Age was not significantly different between the rural and urban samples (*t*_73.42_ = 1.34, *p* = 0.185, Cohen’s d = 0.31).

Independent samples *t*-tests revealed no significant effect of population (i.e., rural/urban) on weight preferences in male faces (*t*_106_ = −0.75, *p* = 0.454, Cohen’s d = 0.15) but a significant effect of population on weight preferences in female faces (*t*_106_ = 3.56, *p* < 0.01, Cohen’s d = 0.69), with the rural participants preferring heavier female faces.

Weight and height were higher among male participants from the urban area (*M*_weight_ = 157.17 lbs., SD = 45.52; *M*_height_ = 67.84 in. SD = 1.96) than male participants from the rural area (*M*_weight_ = 120.78 lbs., SD = 21.53; *M*_height_ = 59.86 in. SD = 7.15) but their BMIs did not differ significantly (*t*_41_ = 0.238, *p* = 0.813, Cohen’s d = 0.07). Weight and height were higher among female participants from the urban area (*M*_weight_ = 125.82 lbs., SD = 25.73; *M*_height_ = 61.21 in. SD = 3.25) than female participants from the rural area (*M*_weight_ = 113.72 lbs., SD = 22.90; *M*_height_ = 56.33 in. SD = 5.85) but their BMIs did not differ significantly (*t*_32.40_ = 1.45, *p* = 0.157, Cohen’s d = 0.51).

### El Salvador

Adiposity preferences were calculated as above. Since independent samples *t*-tests revealed no significant effect of sex of participant on preferences (*p* > 0.212 for all analyses), for all subsequent analyses, data from male and female participants were aggregated. One-sample *t*-tests revealed that the faces selected were significantly different from chance in both the rural and urban samples (*p* < 0.011 for all analyses). Age was not significantly different between the rural and urban samples (*t*_150_ = 0.133, *p* = 0.895, Cohen’s d = 0.02).

Independent samples *t*-tests revealed no significant effect of population (i.e., rural/urban) on weight preferences in male faces (*t*_150_ = 0.77, *p* = 0.445, Cohen’s d = 0.13) but a significant effect of population on weight preferences in female faces (*t*_150_ = 5.91, *p* < 0.001, Cohen’s d = 0.97), with the rural participants preferring heavier female faces.

Weight and height were higher among male participants from the urban area (*M*_weight_ = 174.07 lbs., SD = 35.07; *M*_height_ = 68.97 in. SD = 2.29) than male participants from the rural area (*M*_weight_ = 124.43 lbs., SD = 13.23; *M*_height_ = 64.33 in. SD = 2.05) and their BMIs differed significantly (*t*_39.32_ = −4.96, *p* < 0.001, Cohen’s d = 1.58), with the urban participants having higher BMIs than the rural participants. Weight and height were higher among female participants from the urban area (*M*_weight_ = 132.32 lbs., SD = 26.47; *M*_height_ = 63.09 in. SD = 1.96) than female participants from the rural area (*M*_weight_ = 114.27 lbs., SD = 16.43; *M*_height_ = 59.81 in. SD = 2.14) but their BMIs did not differ significantly (*t*_72.70_ = −1.04, *p* = 0.305, Cohen’s d = 0.24).

## Results: Study 2

Participants showed high levels of inter-rater reliability for all judgments of male and female faces (all Cronbach’s ɑ > 0.97) and we therefore averaged participants’ ratings to produce a mean rating of apparent weight. We then analyzed the data with independent samples *t*-tests with population (i.e., rural/urban) as the grouping variable. A Levene’s correction was used when equal variances could not be assumed.

### Malaysia

Perceived weight ratings were not significantly different between the rural and the urban populations for the male faces (*t*_40.07_ = 0.32, *p* = 0.754, Cohen’s d = 0.10) but they were significantly different for the female faces (*t*_62_ = 3.13, *p* < 0.01, Cohen’s d = 0.80). The female faces from the rural area were rated as heavier than the female faces from the urban area.

### El Salvador

**Fig. 2:**
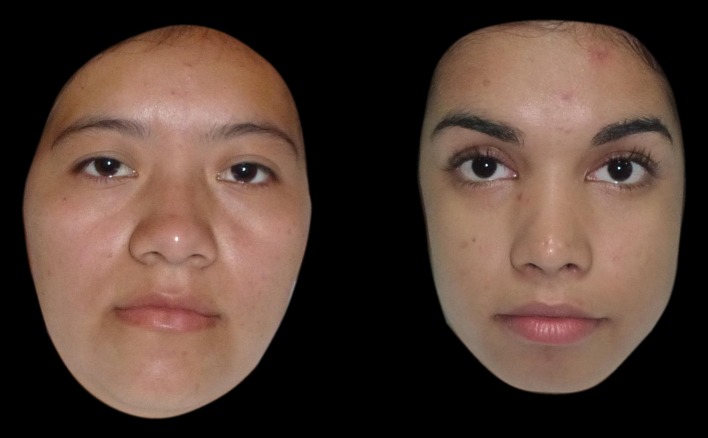
Example of BMI-matched individuals from the rural and urban areas. Two of the female individuals from El Salvador. One is from the rural area (*left*) and one is from the urban area (*right*). Although both individuals have the same BMI (21.2 kg/m^2^), the woman from the rural area (*left*) is perceived to be more overweight than the woman from the urban area (*right*)

Perceived weight ratings were not significantly different between the rural and the urban populations for the male faces (t_67_ = −0.56, *p* = 0.579, Cohen’s d = 0.14) but they were significantly different for the female faces (*t*_81_ = 3.67, *p* < 0.001, Cohen’s d = 0.82). The female faces from the rural area were rated as heavier than the female faces from the urban area (Fig. 2).

### Adiposity Preferences and Perceived Weight Ratings

Partial correlations were run between the adiposity preferences of the participants in Study 1 and their perceived weight ratings as assigned to them by the participants in Study 2, while controlling for the sex of the participants in Study 1 and whether they were from an urban or a rural area. There were no significant correlations in either Malaysia (*p* > 0.106 for all analyses) or El Salvador (*p* > 0.215 for all analyses).

## Discussion

Our results showed no significant relationships between the adiposity preferences of individuals and their perceived weight ratings as assigned to them by other individuals. Our results also showed that there were no differences in adiposity preferences in male faces between the participants from the rural and urban areas in either Malaysia or El Salvador. In addition, we found no differences in the perceived weight ratings between the male faces from the rural and urban areas in both Malaysia and El Salvador.

On the other hand, our results showed that the participants from the rural areas preferred female faces with higher levels of adiposity than the participants from the urban areas in both Malaysia and El Salvador. Additionally, even though the BMIs of women from the urban and rural areas did not differ significantly, we found that the females from the rural areas were rated as looking significantly more overweight than the females from the urban areas in both Malaysia and El Salvador. The effect sizes of population (i.e., rural/urban) on adiposity preferences and perceived weight ratings in female faces were large for both Malaysia and El Salvador.

One possible explanation for such findings is that people from urban areas may store fat differently than people from rural areas. Indeed, individuals vary in the way fat is distributed (Santosa and Jensen 2008). For example, “apple-” and “pear-shaped” bodies are frequently distinguished (Wingard 1990). Individuals with a more apple-shaped body have a higher proportion of visceral body fat and a higher waist girth for any given body mass index. Conversely, a more pear-shaped body is associated with greater fat deposition below the waist. Fat deposition in the cheeks and neck is related to visceral adiposity (Levine et al. 1998; Onat et al. 2009). Individuals with heavier-looking (chubbier) faces are more likely to have apple-shaped bodies and to be predisposed to insulin resistance. Adverse environments with attendant stresses can predispose visceral adiposity (Chrousos 2009). Hence, different populations within the same country may vary in facial morphology as a result of environmental influences including stress and/or dietary composition.

Research has found that harsh environments lead to an increased production of cortisol (Evans and English 2002; Flinn and England 1997), a hormone produced in response to stressors (Flinn and England 1997). For example, one study found a positive link between the number of years lived in poverty and nocturnal urinary cortisol levels (Evans and English 2002). Prolonged exposure to cortisol leads to higher fat deposition on the side of the face (Manenschijn et al. 2012), and since rural environments tend to be harsher than urban environments (Batres and Perrett 2014), women in rural areas may store higher levels of fat in their faces. That may explain why the faces of the female participants from the rural areas in both Malaysia and El Salvador were rated as looking heavier than those from the urban areas even though they did not differ significantly in their BMIs.

Storing fat in the face may be an adaptive response for mating since higher weights in women are considered more attractive in harsh environments (Swami and Tovée 2005; Batres and Perrett 2014), and the face is the most important factor when judging attractiveness (Morse et al. 1976). One possibility for the only observable difference being in female faces is that weight has been found to significantly influence reproductive health in women (Brown et al. 2000; Grodstein et al. 1994), but less so in men (Sallmén et al. 2006). This suggests that having higher levels of facial adiposity may confer stronger evolutionary benefits for women.

Our findings are consistent with the hypothesis that familiarity can contribute to the differences found in face preferences between rural and urban populations. It appears that people from rural areas have a different visual diet of faces than people from urban areas in both Malaysia and El Salvador. More specifically, the faces of the women from the rural areas are rated as looking heavier than the faces of the women from the urban areas. Even without the impact of modern media exposure, people in an urban setting may therefore be exposed to more-slender-looking women’s faces. This exposure to faces that appear thinner may therefore contribute to the observed differences in facial preferences. Future research that examines differences between rural and urban populations should thus also examine the facial characteristics that make up such populations.

It is important to note that, in El Salvador, the faces selected by both the rural and urban participants differed significantly from chance, indicating that there are indeed group differences in ideal adiposity. In Malaysia, however, only the urban participants’ selections differed significantly from chance. That the faces selected by the Malaysian rural sample do not significantly differ from chance (i.e., a 50/50 preference) suggests that this sample is less discriminating (i.e., more random) in their adiposity preferences. One possible explanation for this result is that the Malaysian rural sample may have fewer potential mates to choose from than the Malaysian urban sample and therefore it would prove beneficial to be less discriminating in their preferences. In order to further test this possibility, future research would need to examine participants’ perceived availability of partners alongside the participants’ preferences.

Future research is also needed to replicate our findings in a multi-population sample in order to test their generalizability. Past research has found a strong relationship between body mass and perceived facial adiposity (Coetzee et al. 2009; Tinlin et al. 2013) and that people can accurately estimate a person’s weight based on their face alone (Coetzee et al. 2009, 2010). Our results, however, provide evidence that this finding cannot be generalized between subpopulations. This finding has important implications for future research investigating weight preferences through the use of faces.

Another limitation is that we cannot tease apart the influence of familiarity from other factors that influence face preferences between rural and urban populations. For instance, exposure to the media is greater in urban areas than rural areas (Chan and McNeal 2006) and therefore it is difficult to disentangle the everyday familiarity effect from the media familiarity effect. It would be interesting to examine how face preferences change as people migrate between areas that differ in the visual appearance of the population but do not differ in other factors known to influence face preferences (e.g., media: Becker 2004; stress: Batres and Perrett 2017; health: Tovée et al. 2006; population demographics and density: Scott et al. 2014; violence: Borras et al. 2017; Brooks et al. 2011). Such distinctions would help us understand the role that familiarity plays in what it is that we find attractive. Regardless, this study provides new evidence that familiarity may contribute to the differences observed in facial preferences across populations.
